# The influence of anxiety-related emotional disorders and self-esteem on obesity among Chinese children

**DOI:** 10.12688/f1000research.156811.2

**Published:** 2025-07-30

**Authors:** Xin Shi, Saeid Motevalli, Elaine Jing Ying Chang, Yifei Pan, Jing Sun

**Affiliations:** 1 Psychology, UCSI University, Kuala Lumpur, Federal Territory of Kuala Lumpur, 56000, Malaysia; 2 Education Department, Faculty of Social Sciences and Liberal Arts, UCSI University, Kuala Lumpur, Federal Territory of Kuala Lumpur, 56000, Malaysia

**Keywords:** Self-esteem, Obesity, Panic Disorder, Generalized Anxiety Disorder, Separation Anxiety Disorder, Social Anxiety Disorder, School Avoidance, Good health and Wellbeing

## Abstract

**Background:**

Anxiety and obesity can have negative impacts on the health of children. Understanding the relationship between obesity and mental disorders in children and adolescents will help develop effective prevention and intervention strategies.

**Objectives:**

The main aim is to examine the relationship between anxiety-related emotional disorders, self-esteem, and BMI (obesity) among children, as well as determined the role of anxiety-related emotional disorders and self-esteem in predicting childhood obesity in China.

**Methods:**

Utilizing a non-experimental, cross-sectional study design, data were from 136 participants collected through standardized instrument measures from Chinese children: the “Chinese screening for overweight and obesity among school-age children and adolescents” in the “Health Industry Standards of the People’s Republic of China” as the standard for identifying obesity, the Screen for Anxiety-Related Emotional Disorders (SCARED) for assessing anxiety disorder, and the Children’s Self-Esteem Scale (CSES) for evaluating self-esteem. Statistical analysis, including descriptive statistics, Pearson correlation analysis, and multiple regression analyses was performed using SPSS version 26 to determine the predictive relationships between the variables.

**Results:**

Specific anxiety-related emotional disorders—particularly panic disorder and separation anxiety disorder—significantly predicted higher BMI in Chinese children, while generalized anxiety disorder and school avoidance were associated with lower BMI. Self-esteem, contrary to expectations, did not significantly predict obesity.

**Conclusion:**

This study emphasises the necessity of including early psychological screen in childhood obesity prevention, particularly for anxiety symptoms. The findings have practical consequences for schools, doctors, and public health officials. Further research is needed to better understand the mental-physical health relationship in children.

## Introduction

Childhood obesity has emerged as a critical public health issue worldwide, with profound implications for both physical and mental health. In China, the prevalence of obesity among children has been increasing at an alarming rate. ^1^

Psychological factors, including anxiety-related emotional disorders and self-esteem, have been identified as important contributors to obesity. ^2^ Anxiety-related disorders in children can manifest as excessive worry, fear, or nervousness, often leading to unhealthy eating behaviors such as emotional eating, where food is used as a coping mechanism for stress and anxiety. ^3^ Therefore, children with higher levels of anxiety are more likely to have higher body mass index (BMI) scores. ^4, 5^

Similarly, self-esteem, which reflects a child’s overall sense of self-worth, has been shown to influence eating habits and physical activity levels. Children with low self-esteem may resort to overeating as a coping mechanism, further exacerbating weight gain. ^6^ Overweight and obese children tend to have lower self-esteem than their non-overweight peers, and higher BMI predicts worse self-esteem over time. ^7^

Understanding how anxiety-related emotional disorders and self-esteem affect childhood obesity is essential for developing comprehensive interventions that address both the physical and mental health aspects of obesity. By exploring the intricate links between these constructs, researchers and practitioners can gain valuable insights into both psychological factors that may be more effective in reducing childhood obesity rates.

This study aims to contribute to this understanding by exploring the psychological dimensions of obesity, specifically the roles of anxiety-related emotional disorders and self-esteem. The objective of the study is to examine the relationship between anxiety-related emotional disorders, self-esteem, and BMI (obesity) among children, as well as determined the role of anxiety-related emotional disorders and self-esteem in predicting childhood obesity in China. By addressing these factors, the research seeks to inform the development of holistic interventions promoting physical and mental health among Chinese children.

## Methods

### Study design and participants

This study used a non-experimental, cross-sectional research design to examine the relationship between anxiety-related emotional disorders, self-esteem, and obesity among children, as well as to determine the role of anxiety-related emotional disorders and self-esteem in predicting childhood obesity in China. The sample size of 136 participants was established through G*Power analysis, guaranteeing that the study attains adequate statistical power (0.80) at an alpha level of 0.05, which is crucial for identifying significant relationships among variables. While the sample was chosen from Hefei, Anhui Province, and may not be representative of all of China, it gives valued information about urban and peri-urban child populations in eastern China.

Children who met the set criterias were recruited as participants, inclusion criteria are (a) China citizen, (b) 6-12 years old, (c) currently residing in Anhui, Hefei Province, (d) the absence of any diagnosed psychiatric or neurological disorders, (e) willing to participate in this study, (f) signing the consent form by parents.

To make sure that participants are truly informed, the information letter lists the research title, research purpose, benefits and risks of participating in this research, along with the consent form, which was used to protect the rights of participants. Participants were able to give informed consent from their parents. A total of 25 minutes was allotted for the questionnaires regarding obesity, anxiety and self-esteem. Participants were informed that they would remain anonymous while answering the questionnaires and the responses were confidential as well. No incentives were offered, avoiding undue influence. The researchers declare no conflicts of interest related to this study. No external funding was received; the research was self-funded by the authors, ensuring independence in study design, data analysis, and reporting.

### Measures

***Obesity***

The study used the “Chinese screening for overweight and obesity among school-age children and adolescents” from the “Health Industry Standards of the People’s Republic of China” to identify obesity. This standard is recommended by Dang et al. ^8^ for its prioritization in diagnosing obesity in Chinese children. It has been widely adopted in research on obesity screening for children and adolescents in China, proving its effectiveness and reliability. ^9^ According to China’s National Health Council’s standards for children aged 6-18, BMI is evaluated against age- and gender-specific thresholds: Overweight: BMI ≥ P85 threshold but < P95 threshold. Obesity: BMI ≥ P95 threshold.

***Screen for Child Anxiety Related Disorders (SCARED)***

The SCARED tool, closely related to the DSM, consists of 41 items. It effectively distinguishes between the five types of anxiety: Panic disorder, generalized anxiety disorder, separation anxiety disorder, social anxiety disorder, and school avoidance. ^10^ The Chinese version, revised in 2008, is suitable for assessing anxiety symptoms in Chinese children and adolescents aged 6-18 years in China. ^11^ Its strong psychometric properties, including significant validity and reliability across various populations, have been well-documented in scholarly research. ^12^

***The Children’s Self-esteem Scale***

The CSES, developed by Wei Yunhua, was administered to primary and secondary school students in China and has an internal consistency coefficient of 0.69, indicating high reliability. ^13, 14^ It consists of 26 items scored on a 5-point Likert scale, with higher scores indicating higher self-esteem. The CSES includes reverse-scoring items and measures appearance, sports, ability, sense of achievement, discipline, and public morality/helping others. Scores are categorized into three groups: high self-esteem, middle group, and low self-esteem.

### Data analysis

Preliminary analyses included descriptive statistics to summarize the sample characteristics and the main study variables. Pearson correlation analysis was used to explore the relationships between anxiety-related emotional disorders, self-esteem, and obesity. This analysis helped to identify the strength and direction of the associations between these variables, providing insight into how anxiety and self-esteem are related to obesity in children.

Multiple linear regression analysis was used to examine the extent to which anxiety-related emotional disorders and self-esteem predicted obesity. Panic disorder, generalized anxiety disorder, separation anxiety disorder, social anxiety disorder, and school avoidance were entered as independent variables and BMI score was the dependent variable. Beta coefficients, significance levels, and R-squared statistics were reported to describe the relationships between variables. Significance levels were set at p < .05 for all statistical tests.

## Results

### Descriptive statistics

In this study, a random sampling method was employed to survey student counseling centers in Hefei City, Anhui Province. A total of 136 questionnaires were distributed. After removing invalid and incomplete responses, 121 valid questionnaires were collected. Table 1 shows the demographic information of participants. The students had a mean age of 9.68 years (SD = 1.59), with 55.4% boys and 44.6% girls participants.

**Table 1.** Socio-demographic features of the participants (n=136).

Variable	n	%	Mean	SD
**Children age**			9.68	1.59
7	12	8.8		
8	20	14.7		
9	35	25.7		
10	28	20.6		
11	19	14.0		
12	17	12.5		
13	5	3.7		
**Gender**				
Male	67	55.4		
Female	54	44.6		
**Fathers age group**			39.6	4.853
30 and below	5	4.1		
31-40	62	51.2		
41-50	52	43.0		
51 and above	2	1.7		
**Mothers age group**			38.2	4.464
30 and below	6	5		
31-40	68	56.2		
41-50	47	38.8		
**Fathers’ high education level**				
SPM	13	10.7		
STPM	21	17.4		
Diploma	25	20.7		
Bachelor degree	40	33.1		
Master degree	18	14.9		
PhD	1	0.8		
**Mothers high education level**				
SPM	20	16.5		
STPM	19	15.7		
Diploma	32	26.4		
Bachelor degree	34	28.1		
Master degree	12	9.9		
PhD	1	0.8		
**Family monthly income (RMB)**			11601.65	8677.19
5000 and below	7	5.8		
5001-10000	69	57.0		
10001-20000	41	33.9		
20000 and above	4	3.3		

The distribution of BMI weight status and anxiety symptoms in the sample is presented in Table 2. Among the sample, there were 60 non-overweight and non-obese children (49.6%), comprising 28 boys and 32 girls. There were 25 overweight children (20.7%), with 17 boys and 8 girls. The sample included 36 obese children (29.8%), consisting of 22 boys and 14 girls. The distribution of anxiety disorders among the children was as follows: 25 children (20.7%) had panic disorder, including 12 boys and 13 girls. Generalized anxiety disorder was present in 14 children (11.6%), with 7 boys and 7 girls. Separation anxiety disorder affected 41 children (33.9%), consisting of 22 boys and 19 girls. Social anxiety disorder was found in 20 children (16.5%), including 6 boys and 14 girls. School avoidance was observed in 20 children (14.0%), with 9 boys and 8 girls. In terms of self-esteem, 20 children (16.5%) had low self-esteem, including 11 boys and 9 girls. A moderate level of self-esteem was reported in 88 children (72.7%), comprising 50 boys and 38 girls. High self-esteem was noted in 13 children (10.7%), with 6 boys and 7 girls.

**Table 2.** BMI weight status, anxiety disorder symptom dimensions, and Self-esteem dimensions of Chinese children.

	Male		Female		Total	
	n	%	n	%	n	%
**BMI weight status**						
Non-overweight and non-obesity	28	41.8	32	59.3	60	49.6
Overweight	17	25.4	8	14.8	25	20.7
Obesity	22	32.8	14	25.9	36	29.8
**Anxiety disorder symptom dimension**						
Panic Disorder	12	17.9	13	24.1	25	20.7
Generalized Anxiety Disorder	7	10.4	7	13.0	14	11.6
Separation Anxiety Disorder	22	32.8	19	35.2	41	33.9
Social Anxiety Disorder	6	9.0	14	25.9	20	16.5
School Avoidance	9	13.4	8	14.8	20	14.0
**Self-esteem dimension**						
Low self-esteem	11	16.4	9	16.7	20	16.5
Middle group	50	74.6	38	70.4	88	72.7
High self-esteem	6	9.0	7	13.0	13	10.7

### Reliability analysis

Reliability analysis of the anxiety and self-esteem scales yielded the following results: The anxiety scale, comprising 41 items, had a Cronbach’s α of 0.915, with a mean of 17.56 (SD = 12.520) which indicated very good reliability. The self-esteem scale, comprising 26 items, had a Cronbach’s α of 0.835, with a mean of 71.68 (SD = 14.312) which indicated good reliability ( Table 3).

**Table 3.** Reliability analysis of Anxiety, Self-Esteem Scale.

Scale	No. of item	Cronbach’s alpha	Mean	SD
**Anxiety**	41	0.915	17.56	12.520
**Self-Esteem **	26	0.835	71.68	14.312

### Correlation analysis

Pearson correlation coefficient analysis is conducted to examine the relationship between variables. The research objective is to examine the relationship between anxiety-related emotional disorders, self-esteem, and BMI (obesity) among children. Correspondingly, the hypothesis 1 is: there are significant relationships between anxiety-related emotional disorders, self-esteem, and BMI (obesity).

Table 4 shows the correlation analysis results of each variable. Correlation analysis reveals significant relationships between several variables. BMI shows a significant positive correlation with Panic Disorder (r=0.214*) and Separation Anxiety Disorder (r=0.223*), indicating that higher levels of these disorders are associated with higher BMI. Panic Disorder exhibits strong significant positive correlations with Generalized Anxiety Disorder (r=0.692**), Separation Anxiety Disorder (r=0.557**), Social Anxiety Disorder (r=0.399**), School Avoidance (r=0.520**), and Self-esteem (r=0.407**). These findings suggest that higher levels of one anxiety-related disorder are often accompanied by higher levels of other disorders and lower self-esteem. These correlations support Hypothesis 1 by demonstrating significant relationships between anxiety-related emotional disorders, self-esteem, and BMI scores.

**Table 4.** The correlation analysis results of each variable (N=121).

Variables	1	2	3	4	5	6	7
1 BMI	1.000						
2 Panic disorder	0.214 *	1.000					
3 Generalized Anxiety Disorder	0.055	0.692 **	1.000				
4 Separation Anxiety Disorder	0.223 *	0.557 **	0.623 **	1.000			
5 Social Anxiety Disorder	0.053	0.399 **	0.530 **	0.448 **	1.000		
6 School Avoidance	-0.028	0.520 **	0.473 **	0.443 **	0.298 **	1.000	
7 Self-esteem	0.096	0.407 **	0.553 **	0.431 **	0.396 **	0.437 **	1.00

* Correlation is significant at the 0.05 level (2-tailed).

** Correlation is significant at the 0.01 level (2-tailed).

### Regression analysis

Table 5 presents the multiple linear regression analysis results, examining whether anxiety-related emotional disorders and self-esteem can significantly predict children’s obesity (BMI). The research objective is to determined the role of anxiety-related emotional disorders and self-esteem in predicting childhood obesity in China. Correspondingly, hypothesis 2 of the study is: Anxiety-related emotional disorders and self-esteem can significantly predict obesity among children. The analysis, with BMI as the dependent variable, provides valuable insights into the predictors of BMI among children. Panic Disorder emerged as a significant predictor of higher BMI (B=0.597, p<0.01), indicating that increased levels of panic disorder are associated with higher BMI scores. Similarly, Separation Anxiety Disorder significantly predicts higher BMI (B=0.463, p<0.05), implying that higher levels of separation anxiety disorder are linked to increased BMI.

**Table 5.** Multiple linear regression for emotional anxiety-related emotional disorders and self-esteem as the predictor variable of BMI.

	B	SE	Beta	t	p
Panic Disorder	0.597	0.218	0.351	2.733	<.01 **
Generalized Anxiety Disorder	-0.460	0.223	-0.298	-2.061	<.05 *
Separation Anxiety Disorder	0.463	0.193	0.282	2.402	<.05 *
Social Anxiety Disorder	-0.051	0.177	-0.030	-0.286	0.776
School Avoidance	-0.947	0.436	-0.234	-2.172	<.05 *
Self-esteem	0.046	0.045	0.111	1.015	0.312
R Square = .137, Adjusted R Square = .091					

*B* unstandardized coefficient, *SE* standard error.

* *p* < .05.

** *p* < .01.

Conversely, Generalized Anxiety Disorder significantly predicts a lower BMI (B=-0.460, p<0.05), suggesting that higher levels of generalized anxiety disorder are associated with lower BMI scores. Additionally, School Avoidance is a significant predictor of lower BMI (B=-0.947, p<0.05), indicating that higher levels of school avoidance are associated with lower BMI scores. However, Social Anxiety Disorder and Self-esteem do not significantly predict BMI in this model.

Hypothesis 2 is partially supported. Despite various anxiety-related emotional disorders significantly predicted childhood obesity, social anxiety disorder and self-esteem did not exhibit significant predictive effects. This suggests that although psychological factors influence obesity, their impact may vary in strength and direction. The overall regression model explains 13.7% of the variance in BMI (R Square = 0.137, Adjusted R Square = 0.091), indicating that while these emotional disorders contribute to variations in BMI, a large proportion of the variance remains unexplained.

## Conclusion and Discussion

### Discussion

The first aim of the current study is to examine the relationship between anxiety-related emotional disorders, self-esteem, and BMI (obesity) among children in Hefei, China. The results of the current study showed a significant relationship between anxiety-related emotional disorders, self-esteem, and BMI (obesity).

This finding is consistent with most previous studies, such as Rofey et al., ^15^ who found that anxiety disorders are common among obese children and that both childhood depression and anxiety are associated with increasing BMI percentiles over time. Similarly, Patalay and Hardman ^16^ observed that the association and interrelationship between BMI and internalized symptoms strengthen as children age. Research suggests a significant association between obesity and low self-esteem in Chinese children, with obese primary school children in China having lower self-esteem scores than their normal-weight peers. ^17^ This association is particularly pronounced in girls, with overweight girls showing higher scores for depression symptoms. Body dissatisfaction partially explains the association between obesity and low self-esteem in both sexes. ^18^ The relationship between obesity and self-esteem appears to strengthen as children progress through primary school. ^19^ While some studies suggest that low self-esteem may precede the development of obesity, ^20^ others suggest that obesity is more likely to lead to low self-esteem, ^21^ highlighting the need for early intervention in the management of childhood obesity.

The current study also examined the role of anxiety-related emotional disorders and self-esteem in predicting childhood obesity in Hefei, China. Multiple regression analysis revealed that panic disorder and separation anxiety disorder significantly predicted higher BMI, indicating that children with these conditions were more likely to be obese. This is consistent with the findings of Haghighi et al., ^22^ who reported that children and adolescents with Panic Disorder had the highest mean BMI. Similarly, pre-adolescent Separation Anxiety symptoms are associated with increased BMI in adolescent boys, with the relationship mediated by emotional eating, hunger sensitivity, and difficulty regulating emotions. ^23^ In addition, early separation anxiety and a focused attachment style are significant predictors of body dissatisfaction in women with eating disorders, which may increase the risk of developing eating disorders.

In contrast, generalized anxiety disorder and school avoidance significantly predict lower BMI, meaning that children may be underweight, probably due to reduced appetite or avoidance behaviors that affect eating patterns. One study shows that Generalized Anxiety Disorder is the most common among school-aged children in China. ^24^

Notably, social anxiety disorder and self-esteem did not significantly predict BMI in this study, which is inconsistent from certain prior findings, possibly due to their complex or indirect effects not captured by the analysis. Despite the extensive argument in the prior literature that low self-esteem and increased social anxiety are risk factors for obesity, ^25^ results of the curent study challenge this assumption. It is important to note that similar findings have been reported elsewhere, suggesting that social anxiety and self-esteem may not consistently predict BMI or obesity among children. For instance, Wang et al. ^26^ found that childhood obesity predicts lower self-esteem, whereas self-esteem does not predict obesity. Besides, Wang et al. ^27^ argued that obesity was negatively impact children’s self-esteem; however, self-esteem does not influence obesity or academic performance among children. In terms of social anxiety disorder, Ozkan et al. ^28^ discovered that there is no correlation between BMI and depression/social anxiety scores in obese children suggesting that the influence of social anxiety on obesity may be contingent upon contextual factors or moderated by additional variables, including peer relationships or social support systems. Besides, Anderson et al. ^29^ indicated that adolescent obesity in males did not show a significant correlation with a higher likelihood of developing major depressive disorder or anxiety disorder. Hence, findings of the study indicated that social anxiety and self-esteem were not significant predictors of obesity, contradicting the widely held belief that higher anxiety and lower self-esteem contributes to weight gain through emotional eating and decreased physical activity.

Other literature suggesting that social appearance anxiety is negatively correlated with self-esteem, with BMI acting as a mediating factor. ^30^ Kornapalli et al. ^25^ found that body image concerns were negatively correlated with self-esteem and positively correlated with social anxiety, with significant BMI-related differences. Furthermore, Horenstein et al. ^31^ argued that Social Anxiety moderates the relationship between BMI and exercise avoidance motivation, particularly for those with higher BMIs. Interestingly, Ternouth et al. ^32^ found that childhood emotional difficulties were predictive of weight gain in women but did not have the same effect in men, while low childhood self-esteem predicted weight gain in both men and women, with a stronger effect in women. In contrast, Roberts and Duong ^33^ found that anxiety disorders may increase risk of obesity in adolescent males, but not females. While these studies demonstrate associations between social anxiety, self-esteem, and BMI, they do not definitively establish the specific predictive value of social anxiety and self-esteem for BMI.

Furthermore, Sheinbein et al. ^34^ mention in their study that the pathology of eating disorders in children is significantly correlated with anxiety symptoms in children. Children with obesity or eating disorders often have difficulty controlling their eating behavior, which leads to weight gain and associated negative emotions such as shame, guilt, and anxiety. In addition, T. Wang et al. ^35^ emphasized the importance of healthy weight status for the development of young children, particularly in terms of cognitive function. Moss et al. ^36^ mentioned that obesity may impair the activation of neurophysiological components that are essential for cognitive function, thus affecting children’s cognitive function. These studies again highlight the link between cognitive development in obese children and emotional problems such as anxiety and low self-esteem. For this matter, numerous studies have demonstrated that physical activity interventions have the potential to enhance psychological outcomes in obese youth, including improved body image, increased self-esteem, and reduced depression. ^37, 38^ In addition, early intervention is critical, with overweight/obese youth receiving targeted mental health screening in early adolescence for depressive and adjustment disorders, and in early childhood for anxiety disorders. ^39^ Consequently, it is imperative to incorporate psychological interventions that address mental health issues into obesity prevention and treatment programs for children and adolescents, focusing on both physical and psychological well-being to effectively improve health outcomes.

### Limitation

However, some limitations of the study should be noted. First of all, the cross-sectional, non-experimental design of the study makes it difficult to establish causal relationships between self-esteem, anxiety-related emotional disorders, and obesity. The observed associations may have been influenced by other factors, such as parental influence, lifestyle habits, and environmental conditions, as the data were collected at a single point in time. In order to gain a more comprehensive understanding of the direction of these relationships, future research should consider experimental designs that enable controlled interventions as well as longitudinal studies that monitor changes over time.

Secondly, the study depended exclusively on self-reported measures, which may be influenced by biases such as social desirability and inaccurate recall. Participants’ symptoms may have been underreported or overestimated, compromising the reliability of the findings. For this matter, future studies should use more objective assessment tools, such as clinical evaluations, behavioural observations, or physiological markers, alongside self-report questionnaires, to gain a more comprehensive and in-depth understanding of psychological and physical health outcomes.

Thirdly, the sample size is relatively small, and the focus on a single province limits the findings’ generalisability. Psychological health and obesity-related factors can vary by region in China, particularly between urban and rural areas with different socioeconomic and educational resources. Hence, expanding the sample to include multiple provinces or a nationally representative cohort would result in more robust and generalisable findings.

Lastly, although this study identifies significant correlations between anxiety-related disorders, self-esteem, and obesity, it does not thoroughly investigate the mechanisms underlying these associations. In order to better understand how these variables interact, future research should look into potential mediators and moderators, such as coping strategies, family dynamics, and peer influences. Furthermore, studies of interventions that assess the efficacy of behavioural or psychological programs may offer useful information about methods for enhancing children’s mental health and weight-related outcomes.

### Implication

The study’s findings have important implications for school and community, public health policymakers, mental health and healthcare professionals, as well as future researchers.

Schools and community organisations should provide structured psychological support and health education to assist children in developing better coping mechanisms and healthier habits. Specifically, early intervention programs for children should be designed to boost self-esteem, reduce anxiety, and promote healthy lifestyle habits. Besides, workshops and parental guidance sessions can provide families with the resources to tackle both emotional well-being and physical health in their children.

For public health policymakers, it should be a priority to integrate mental health initiatives with obesity prevention strategies. The resources necessary for children to maintain both their physical health and emotional resilience can be provided by policies that support school-based mental health services, accessible counselling programs, and community-based health promotion campaigns. Moreover, policymakers should strive to reduce the barriers to mental health care by increasing the availability of affordable psychological services.

Mental health and healthcare professionals should adopt a multidisciplinary approach that integrates mental health counselling with lifestyle interventions. In this regard, educational institutions, paediatricians, and mental health practitioners have to cooperate to develop intervention programs that tackle psychological issues and maladaptive behaviours, thereby ensuring an effective treatment strategy.

Lastly, this study has provided future researchers with scientific information regarding the impact of anxiety-related emotional disorders and self-esteem on obesity in children. This information will enable them to conduct further longitudinal studies and experimental designs in order to elucidate the causal pathways between anxiety, self-esteem, and obesity. In addition, future research should investigate mediators including familial influence, peer relationships, and socioeconomic variables to formulate targeted interventions that more effectively address the psychological and physical health needs of children.

### Summary

This study highlights significant relationships between anxiety-related emotional disorders, self-esteem, and obesity among children in Hefei City, Anhui Province. Findings indicates that specific anxiety-related emotional disorders—particularly panic disorder and separation anxiety disorder—significantly predicted higher BMI in Chinese children, while generalized anxiety disorder and school avoidance were associated with lower BMI. Self-esteem, contrary to expectations, did not significantly predict obesity. The results highlight the need for early psychological screening and intervention as part of obesity prevention programs, particularly targeting anxiety symptoms in school-aged children. Despite the study’s limitations, it provides a valuable foundation for future research and offers insights for clinical practice, educational settings, and public health policies. Continued efforts to understand and address the interplay between mental health and obesity are crucial for promoting children’s overall well-being.

## Ethical considerations

The study adhered to ethical guidelines, securing approval from the institutional review board (IRB), Department of Psychology, UCSI University, Ethical Clearance Application, DEC/PSY/2022/09/24/03 on 28/03/2024 and because of the age of children (minors under 16 years) the written informed consent from all participants’ parents or guardians was collected. Confidentiality and anonymity were assured, and participation was voluntary. Data were used solely for research purposes, maintaining participants’ anonymity. Participants were informed of their right to withdraw from the study at any time, ensuring their autonomy and comfort throughout the research process.

## Author contributions

All of the authors greatly aided the manuscript’s development.

**Table:** 

Contributor role	Authors
Conceptualization	Dr. Saeid **Motevalli**
Data Curation	Xin **Shi**
Formal Analysis	Xin **Shi**
Funding Acquisition	Xin **Shi**
Investigation	Xin **Shi**
Methodology	Dr. Saeid **Motevalli**
Project Administration	Xin **Shi**
Resources	Xin **Shi**
Software	Elaine Jing Ying **Chang**
Supervision	Dr. Saeid **Motevalli**
Validation	Elaine Jing Ying **Chang**
Visualization	Xin **Shi**
Writing – Original Draft Preparation	Xin **Shi**
Writing – Review & Editing	Dr. Saeid **Motevalli**
Writing – Review & Editing	Yifei **Pan**
Writing – Review & Editing	Jing **Sun**

## Data Availability

Figshare: The Influence of Anxiety-Related Emotional Disorders and Self-esteem on Obesity Among Chinese Children. https://doi.org/10.6084/m9.figshare.27968115.v1. ^40^ The project contains the following underlying data:
• Datasets.xlsx. (Anonymised answers to the questionnaire) Data are available under the terms of the Creative Commons Attribution 4.0 International license (CC-BY 4.0). Figshare: The Influence of Anxiety-Related Emotional Disorders and Self-esteem on Obesity Among Chinese Children. https://figshare.com/articles/figure/Extended_data/27036154.v1. ^41^
• English-Demographic Information (Blank English version of the questionnaire) Data are available under the terms of the Creative Commons Attribution 4.0 International license (CC-BY 4.0).
• English-Screen for Child Anxiety-Related Disorders (SCARED) (Blank English version of the questionnaire) Data are available under the terms of the Creative Commons Attribution 4.0 International license (CC-BY 4.0).
• English- The children’s self-esteem scale (Blank English version of the questionnaire) Data are available under the terms of the Creative Commons Attribution 4.0 International license (CC-BY 4.0).
• Chinese-Demographic Information (Blank Chinese version of the questionnaire) Data are available under the terms of the Creative Commons Attribution 4.0 International license (CC-BY 4.0).
• Chinese-Screen for Child Anxiety-Related Disorders (SCARED) (Blank Chinese version of the questionnaire) Data are available under the terms of the Creative Commons Attribution 4.0 International license (CC-BY 4.0).
• Chinese- The children’s self-esteem scale (Blank Chinese version of the questionnaire) Data are available under the terms of the Creative Commons Attribution 4.0 International license (CC-BY 4.0).

## References

[1] Organization, W.H: Obesity: preventing and managing the global epidemic: report of a WHO consultation. 2000. 11234459

[2] Beesdo K Knappe S Pine DS : Anxiety and anxiety disorders in children and adolescents: developmental issues and implications for DSM-V. *Psychiatr. Clin.* 2009;32(3):483–524. 10.1016/j.psc.2009.06.002 PMC3018839 19716988

[3] Masood B Moorthy M : Causes of obesity: a review. *Clin. Med.* 2023;23(4):284–291. 10.7861/clinmed.2023-0168 37524429 PMC10541056

[4] Suzuki Y : The association between obesity and hyperactivity/anxiety among elementary school students in Japan. *Int. J. Behav. Med.* 2020;27:79–86. 10.1007/s12529-019-09827-x 31820287

[5] Drosopoulou G : Psychosocial health of adolescents in relation to underweight, overweight/obese status: The EU NET ADB survey. *Eur. J. Pub. Health.* 2021;31(2):379–384. 10.1093/eurpub/ckaa189 33152069

[6] Lindberg L : Anxiety and depression in children and adolescents with obesity: a nationwide study in Sweden. *BMC Med.* 2020;18:1–9. 10.1186/s12916-020-1498-z 32079538 PMC7033939

[7] Joshi S : Self-Esteem: Relationship with Age, Gender and BMI in Adolescents. *Indian Journal of Youth and Adolescent Health.* 2023.10(2): p.1–7. (E-ISSN: 2349-2880).

[8] Dang J-J : Methods for evaluating overweight and obesity among children and adolescents and application in SPSS and SAS. *Zhonghua yu Fang yi xue za zhi [Chinese Journal of Preventive Medicine].* 2022;56(1):75–81. 10.3760/cma.j.cn112150-20210319-00271 35092995

[9] Tian T : Multilevel Analysis of the Nutritional and Health Status among Children and Adolescents in Eastern China. *Nutrients.* 2022;14(4):758. 10.3390/nu14040758 35215409 PMC8877382

[10] DeSousa DA : Sensitivity and specificity of the Screen for Child Anxiety Related Emotional Disorders (SCARED): a community-based study. *Child Psychiatry Hum. Dev.* 2013;44:391–399. 10.1007/s10578-012-0333-y 22961135

[11] Su L : Reliability and validity of the screen for child anxiety related emotional disorders (SCARED) in Chinese children. *J. Anxiety Disord.* 2008;22(4):612–621. 10.1016/j.janxdis.2007.05.011 17628391

[12] Birmaher B : The screen for child anxiety related emotional disorders (SCARED): Scale construction and psychometric characteristics. *J. Am. Acad. Child Adolesc. Psychiatry.* 1997;36(4):545–553. 10.1097/00004583-199704000-00018 9100430

[13] Wu Y-L : Change and associated factors of self-esteem among children in rural China: A two-year longitudinal study. *Psychol. Health Med.* 2015;20(8):879–888. 10.1080/13548506.2014.983136 25410380

[14] Li P : A Case Study on the Relationship between Children’s Self-Esteem and Parental Rearing Style. 2019.

[15] Rofey DL : A longitudinal study of childhood depression and anxiety in relation to weight gain. *Child Psychiatry Hum. Dev.* 2009;40:517–526. 10.1007/s10578-009-0141-1 19404733 PMC2918233

[16] Patalay P Hardman CA : Comorbidity, codevelopment, and temporal associations between body mass index and internalizing symptoms from early childhood to adolescence. *JAMA Psychiatry.* 2019;76(7):721–729. 10.1001/jamapsychiatry.2019.0169 30892586 PMC6583661

[17] Zhang J : Prevalence and stabilizing trends in overweight and obesity among children and adolescents in China, 2011-2015. *BMC Public Health.* 2018;18:1–7. 10.1186/s12889-018-5483-9 PMC5930802 29716560

[18] Li Y-P : Report on childhood obesity in China (5) body weight, body dissatisfaction and depression symptoms of Chinese children aged 9-10 years. *Biomed. Environ. Sci.* 2007;20(1):11–18. 17458136

[19] Hesketh K Wake M Waters E : Body mass index and parent-reported self-esteem in elementary school children: evidence for a causal relationship. *Int. J. Obes.* 2004;28(10):1233–1237. 10.1038/sj.ijo.0802624 15314637

[20] French SA Story M Perry CL : Self-esteem and obesity in children and adolescents: a literature review. *Obes. Res.* 1995;3(5):479–490. 10.1002/j.1550-8528.1995.tb00179.x 8521169

[21] Öz B Kıvrak AC : Evaluation of depression, anxiety symptoms, emotion regulation difficulties, and self-esteem in children and adolescents with obesity. *Arch. Pediatr.* 2023;30(4):226–231. 10.1016/j.arcped.2023.02.003 37062655

[22] Haghighi M : The relation between anxiety and BMI–is it all in our curves? *Psychiatry Res.* 2016;235:49–54. 10.1016/j.psychres.2015.12.002 26708439

[23] Wilkinson LL : Explaining the relationship between attachment anxiety, eating behaviour and BMI. *Appetite.* 2018;127:214–222. 10.1016/j.appet.2018.04.029 29733864

[24] Wang F : Prevalence and comorbidity of anxiety disorder in school-attending children and adolescents aged 6–16 years in China. *BMJ Paediatr. Open.* 2024;8(1):e001967. 10.1136/bmjpo-2023-001967 38538104 PMC10982779

[25] Kornapalli SE : The Relationship between Body shape concern, Self-esteem, Social anxiety and Body mass index in College students. *Telangana J. Psychiatry.* 2017;3(2):78–84.

[26] Wang F Wild TC Kipp W : The influence of childhood obesity on the development of self-esteem. *Health Rep.* 2009;20(2):21–27. 19728582

[27] Wang F Veugelers PJ : Self-esteem and cognitive development in the era of the childhood obesity epidemic. *Obes. Rev.* 2008;9(6):615–623. 10.1111/j.1467-789X.2008.00507.x 18647242

[28] Ozkan EA Gul AI Ozdemir ZT : Evaluation of depression and social anxiety symptoms in obese children. *Dusunen Adam J. Psychiatry Neurol. Sci.* 2016;29(1):8–14. 10.5350/DAJPN2016290101

[29] Anderson SE Cohen P Naumova EN : Adolescent obesity and risk for subsequent major depressive disorder and anxiety disorder: prospective evidence. *Psychosom. Med.* 2007;69(8):740–747. 10.1097/PSY.0b013e31815580b4 17942847

[30] Göbel P : Social appearance anxiety and self-esteem in women: could body mass index have a mediating role? *Behavioral Psychology=Psicología Conductual.* 2023;31(1):25–37. 10.51668/bp.8323102n

[31] Horenstein A : Social anxiety moderates the relationship between body mass index and motivation to avoid exercise. *Body Image.* 2021;36:185–192. 10.1016/j.bodyim.2020.11.010 33360475

[32] Ternouth A Collier D Maughan B : Childhood emotional problems and self-perceptions predict weight gain in a longitudinal regression model. *BMC Med.* 2009;7:1–9. 10.1186/1741-7015-7-46 19747369 PMC2753342

[33] Roberts RE Duong HT : Do anxiety disorders play a role in adolescent obesity? *Ann. Behav. Med.* 2016;50(4):613–621. 10.1007/s12160-016-9786-8 26961207

[34] Sheinbein DH : Factors associated with depression and anxiety symptoms among children seeking treatment for obesity: A social-ecological approach. *Pediatr. Obes.* 2019;14(8):e12518. 10.1111/ijpo.12518 30990254 PMC7081722

[35] Wang T : Association between dietary patterns and cognitive ability in Chinese children aged 10–15 years: Evidence from the 2010 China Family Panel Studies. *BMC Public Health.* 2021;21:1–13. 10.1186/s12889-021-12209-2 34863128 PMC8642971

[36] Moss S : Overweight/obesity and socio-demographic disparities in children’s motor and cognitive function. *Front. Psychol.* 2023;14:1134647. 10.3389/fpsyg.2023.1134647 37287792 PMC10242128

[37] Topçu S Orhon FŞ Tayfun M : Anxiety, depression and self-esteem levels in obese children: a case-control study. *J. Pediatr. Endocrinol. Metab.* 2016;29(3):357–361. 10.1515/jpem-2015-0254 26565543

[38] Vila G Zipper E Dabbas M : Mental disorders in obese children and adolescents. Psychosom. Med.,2004;66(3):387–394. 10.1097/01.psy.0000126201.12813.eb 15184702

[39] Carroll R Bice AA Roberto A : Examining mental health disorders in overweight and obese pediatric patients. *J. Pediatr. Health Care.* 2022;36(6):507–519. 10.1016/j.pedhc.2022.05.017 35760667

[40] Motevalli S : V4_05 Dec 2024 data.sav.Dataset. *figshare.* 2024. 10.6084/m9.figshare.27968115.v1

[41] Shi X : Extended data. figshare. *Figure.* 2024. 10.6084/m9.figshare.27036154.v1

