# Supplementary material for: The influence of anxiety-related emotional disorders and self-esteem on obesity among Chinese children
Source: F1000Res. 2026 May 5;14:84. Originally published 2025 Jan 15. [Version 3] doi: 10.12688/f1000research.156811.3 (PMC13197749; doi:10.12688/f1000research.156811.3)
Supplement: Peer review history

# Reviewer report: Reviewer response for version 3

**DOI:** 10.5256/f1000research.200432.r485809

**Published:** 2026-05-22

**Authors:** Tatiana Palotta Minari

**Affiliations:** 1 Federal University of São Paulo (UNIFESP), Santos, Brazil

*1. Overall Assessment*

 This manuscript investigates the relationship between anxiety-related emotional disorders, self-esteem, and obesity among Chinese children. The study is relevant and timely, given the rising prevalence of childhood obesity and the growing recognition of psychological factors in its etiology. The authors have strengthened the methodology in this revised version, improving clarity and rigor. The findings contribute to the literature by highlighting specific anxiety disorders (panic and separation anxiety) as predictors of higher BMI, while generalized anxiety and school avoidance are linked to lower BMI.

*2. Strengths*

- Clear research objectives: The study is well-focused on examining psychological predictors of obesity.
- Methodological improvements: The inclusion of sample size calculation, pilot study, and regression assumption checks enhances credibility.
- Use of validated instruments: The SCARED and CSES scales are appropriate and reliable for the Chinese context.
- Balanced findings: The nuanced results (different anxiety disorders predicting BMI in opposite directions) add depth to the discussion.
- Public health relevance: The study emphasizes the importance of early psychological screening in obesity prevention.

*3. Major revisions*

- Sample representativeness: The sample was drawn from counseling centers, which may introduce bias. This limitation should be discussed more explicitly in the conclusion.
- Statistical power: Although the sample size exceeded the minimum requirement, the explained variance in BMI (R² = 0.137) is relatively low. The authors should acknowledge that other unmeasured factors likely play a larger role.
- Self-esteem findings: The non-significant role of self-esteem deserves deeper exploration. Why might self-esteem not predict BMI in this sample, despite prior literature suggesting otherwise?
- Discussion: The discussion could be expanded to compare findings with international studies and to explore cultural factors that may influence the observed relationships.
- Language and style: Minor grammatical issues and occasional awkward phrasing should be corrected for smoother readability.

*4. Minor revisions*

- Ensure consistent formatting of statistical symbols (e.g., p < .05 vs. p < 0.05).
- Clarify whether BMI categories (overweight, obesity) were analyzed separately or only as continuous BMI scores.
- Some tables (e.g., Table 1) contain redundant demographic details that could be streamlined.

Is the work clearly and accurately presented and does it cite the current literature?

Partly

If applicable, is the statistical analysis and its interpretation appropriate?

Partly

Are all the source data underlying the results available to ensure full reproducibility?

Partly

Is the study design appropriate and is the work technically sound?

Partly

Are the conclusions drawn adequately supported by the results?

Partly

Are sufficient details of methods and analysis provided to allow replication by others?

Partly

Reviewer Expertise:

anxiety-related emotional disorders

I confirm that I have read this submission and believe that I have an appropriate level of expertise to confirm that it is of an acceptable scientific standard, however I have significant reservations, as outlined above.

# Reviewer report: Reviewer response for version 3

**DOI:** 10.5256/f1000research.200432.r481764

**Published:** 2026-05-13

**Authors:** Mahmoud Danaee

**Affiliations:** 1 University of Malaya, Kuala Lumpur, Malaysia

Authors addressed all my comments.

Is the work clearly and accurately presented and does it cite the current literature?

Yes

If applicable, is the statistical analysis and its interpretation appropriate?

Partly

Are all the source data underlying the results available to ensure full reproducibility?

Partly

Is the study design appropriate and is the work technically sound?

Yes

Are the conclusions drawn adequately supported by the results?

Yes

Are sufficient details of methods and analysis provided to allow replication by others?

Partly

Reviewer Expertise:

Research methodology and statistical analysis

I confirm that I have read this submission and believe that I have an appropriate level of expertise to confirm that it is of an acceptable scientific standard.

# Reviewer report: Reviewer response for version 2

**DOI:** 10.5256/f1000research.184521.r405305

**Published:** 2025-09-03

**Authors:** Mahmoud Danaee

**Affiliations:** 1 University of Malaya, Kuala Lumpur, Malaysia

The manuscript mentions “random sampling” only in Results. Sampling procedures should be reported in Methodology. It is recommended to provide more details on how random sampling was conducted (e.g., sampling frame, sampling process and discuss them in more detail to allow the reader to understand the representativeness of the sample and potential sources of bias.

 Description of sample size calculation is not clear enough. The study states that GPower was used to calculate a sample size of 136 but does not mention the specific statistical test or method used within GPower. Since the study conducted a multivariate linear regression analysis, the assumptions made (e.g., type of test, number of predictors, effect size) should have been specified. Without this information, it is unclear if the reported sample size was appropriate for the planned analyses.

 It is not reported whether the instruments used in the study were validated or tested for reliability through a pilot study. Validity and reliability should be reported to ensure that the instruments used accurately measure the intended constructs and provide consistent results.

 Since a multivariate linear regression analysis was conducted, it would be important to report whether the key assumptions of regression (normality of the dependent variable, linearity, homoscedasticity, multicollinearity among predictors) were checked. Checks for normality, linearity, homoscedasticity, and multicollinearity should have been conducted and reported, as violations of these assumptions could impact the validity of the findings.

 It is unclear whether the final regression model included gender, age, or other potential confounding variables. it should be made clear whether these factors were controlled for in the analysis to rule out biased or misleading results.

Is the work clearly and accurately presented and does it cite the current literature?

Yes

If applicable, is the statistical analysis and its interpretation appropriate?

Partly

Are all the source data underlying the results available to ensure full reproducibility?

Partly

Is the study design appropriate and is the work technically sound?

Yes

Are the conclusions drawn adequately supported by the results?

Yes

Are sufficient details of methods and analysis provided to allow replication by others?

Partly

Reviewer Expertise:

Research methodology and statistical analysis

I confirm that I have read this submission and believe that I have an appropriate level of expertise to confirm that it is of an acceptable scientific standard, however I have significant reservations, as outlined above.

# Response

**Published:** 2026-04-27

**Authors:** Saeid Motevalli

**Affiliations:** Psychology, UCSI University, Kuala Lumpur, Federal Territory of Kuala Lumpur, Malaysia

Thank you for your valuable comments. The sampling procedure has been moved to the Methods section and further elaborated to clarify the sampling frame, process, and potential sources of bias .

 Additional details on the G*Power analysis, including the statistical test, effect size, number of predictors, and assumptions, have been clarified in the Methods section to justify the adequacy of the sample size.

 Modifications have been made to the Methods section to strengthen the methodological rigor of the study .

 Information regarding the assessment of regression assumptions, including normality, linearity, homoscedasticity, and multicollinearity, has been added to the data analysis part.

 The manuscript has been revised to clarify that demographic variables such as age and gender were considered but not included in the final regression model is now acknowledged.

 We hope that these revisions have adequately addressed the reviewer’s concerns and improved the clarity and rigor of the manuscript. Thank you again for your insightful feedback.

# Reviewer report: Reviewer response for version 1

**DOI:** 10.5256/f1000research.172179.r365605

**Published:** 2025-02-21

**Authors:** Mohtaram Rabbani

**Affiliations:** 1 Hong Kong University, Hong Kong, China

1. Please add research objectives at the beginning to provide a clear roadmap for the study and guide the reader through the research aims.
2. Please provide a rationale for the sample size of 136 participants and discuss its representativeness for the broader population of Chinese children. Consideration of statistical power and sample adequacy could strengthen the study's findings.
3. Address the limitations of the non-experimental, cross-sectional design by discussing potential confounding variables and alternative explanations for the observed relationships. Consider incorporating longitudinal or experimental designs for future studies.
4. Given the reliability issues identified in the school avoidance scale, consider revising or validating the measurement tools to ensure their accuracy and reliability. Discuss the limitations of self-reported data and potential biases.
5. Provide a more detailed explanation of the statistical analyses conducted, including assumptions, model fit statistics, and potential interactions among variables. Consider exploring additional statistical methods to strengthen the predictive models.
6. Provide a more critical discussion of the study's findings by comparing them to existing literature, addressing inconsistencies, and explaining the implications of the results for theory and practice. Consider discussing the practical significance of the results in addition to statistical significance.
7. While mentioning ethical considerations is important, provide more details on how confidentiality and anonymity were ensured, how participants were recruited, and how potential biases were mitigated. Consider discussing any potential conflicts of interest or funding sources.
8. Offer concrete suggestions for future research directions based on the study's limitations and findings. Propose ways to address the identified issues and further explore the complex relationships between anxiety-related emotional disorders, self-esteem, and obesity.
9. Strengthen the conclusion by summarizing key findings, discussing their implications for clinical practice and policy, and highlighting the significance of the study within the broader context of childhood obesity research. Consider addressing the practical implications for intervention programs.

Is the work clearly and accurately presented and does it cite the current literature?

Partly

If applicable, is the statistical analysis and its interpretation appropriate?

Partly

Are all the source data underlying the results available to ensure full reproducibility?

Partly

Is the study design appropriate and is the work technically sound?

Partly

Are the conclusions drawn adequately supported by the results?

Partly

Are sufficient details of methods and analysis provided to allow replication by others?

Partly

Reviewer Expertise:

NA

I confirm that I have read this submission and believe that I have an appropriate level of expertise to confirm that it is of an acceptable scientific standard, however I have significant reservations, as outlined above.

# Response

**Published:** 2025-07-04

**Authors:** Saeid Motevalli

**Affiliations:** Psychology, UCSI University, Kuala Lumpur, Federal Territory of Kuala Lumpur, Malaysia

Thank you for your insightful feedback. We have made several revisions to address your concerns and improve the manuscript.

 To begin, we have included research objectives at abstract part and the end of introduction part, providing a clear roadmap for the study that was previously missing. Regarding the sample size, we have provided a detailed rationale for the 136 participants, explaining how the size was determined using G*Power analysis to ensure adequate statistical power and representativeness for children in Hefei, Anhui Province.

 We have expanded the discussion on the limitations of the cross-sectional design, addressing potential confounding variables and the need for longitudinal or experimental designs in future studies.

 Additionally, the reliability of the anxiety scale is acceptable mentioned in realiability part, and we acknowledge the limitations of self-reported data, suggesting more objective measures in future research.

 In response to your request, we have clarified the statistical analyses to improve transparency. The Discussion now includes a more critical comparison with existing literature, addressing inconsistencies and practical significance of the results.

 We have also provided more detailed ethical considerations, explaining how confidentiality, anonymity, and informed consent were ensured.

 Moreover, some content of limitations were changed, followed by recommendations accordingly added. Also, an implementation section was added to the article.

 Finally, the Conclusion has been revised to better summarize the study’s key findings and their practical implications for clinical practice and policy.

 These revisions strengthen the manuscript and we hope they address your concerns. Thank you again for your valuable feedback.
